# Multifactorial analysis and construction of a nomogram model for postoperative recurrence of glomus jugulare tumor

**DOI:** 10.3389/fonc.2025.1662079

**Published:** 2025-10-06

**Authors:** Kun Li, Qi Lu, Xiaoyan Guo, Ting Kou, Jiyue Chen, Shiming Yang, Weidong Shen

**Affiliations:** ^1^ Senior Department of Cardiology, the Sixth Medical Center of PLA General Hospital, Beijing, China; ^2^ National Clinical Research Center for Otolaryngologic Diseases, Beijing, China; ^3^ Beijing Key Laboratory of Hearing Impairment Prevention and Treatment, Beijing, China; ^4^ Senior Department of Otolaryngology Head and Neck Surgery, People's Liberation Army General Hospital, Beijing, China

**Keywords:** GJT, nomogram, postoperative recurrence, prognostic factors, AUC

## Abstract

**Background:**

To derive and validate a prognostic nomogram for predicting postoperative recurrence in patients with glomus jugulare tumor(GJT) to assist clinical decision-making.

**Methods:**

A retrospective analysis was conducted on the clinical data of a total of 318 patients diagnosed with GJT at a single tertiary medical center. The study collected information on patient demographics, clinical symptoms and signs, examination results, and the extent of tumor growth. Patients were categorized into two groups based on DFS (Disease - free survival): those who experienced recurrence and those who did not. A nomogram model was developed using logistic regression to analyze the risk of postoperative recurrence.

**Results:**

Multivariate logistic regression analysis identified age, immunohistochemical expression levels of Ki-67 and S-100 and tumor invasion extent were significantly associated as independent predictors. These independent predictors were incorporated into a nomogram. The logistic regression-based nomogram showed excellent predictive accuracy of the nomogram model in the training set, validation set, and test set, with corresponding areas under the curve (AUC) of 0.863, 0.711, and 0.784, respectively.

**Conclusions:**

The nomogram effectively predicts GJT recurrence, validated internally and externally, aiding clinical risk stratification.

## Introduction

Surgical resection is the primary treatment for tympanojugular paragangliomas(TJPGLs), which account for nearly 40% of head and neck paragangliomas (HNPGLs). These tumors may severely affect various cranial nerves and cause significant clinical manifestations. However, the majority of patients achieve long-term disease-free survival following surgery. Studies indicate a recurrence rate of approximately 6.5% within 5 years following definitive treatment, with nearly one-third of patients experiencing recurrence more than 10 years after initial surgery (1, 2).

Currently, neither the NCCN (National Comprehensive Cancer Network) nor CSCO (Chinese Society of Clinical Oncology) guidelines provide dedicated protocols for TJPGLs management. Relevant recommendations are dispersed within broader guidelines for head and neck cancers or neuroendocrine tumors. These guidelines lack clarity on whether adjuvant radiotherapy (RT) reduces recurrence rates in the overall HNPGL population (a point of ongoing debate) and fail to define strategies for identifying high-risk patients or implementing appropriate postoperative interventions. Existing evidence suggests adjuvant RT may prolong DFS in patients with near-total resection or those at high risk of recurrence (3–5).Nevertheless, the potential risks of local discomfort, pharyngeal edema, and neurological injury associated with RT must be considered. Consequently, the precise identification of high-risk recurrence patients for adjuvant RT holds promise for reducing overall recurrence rates while minimizing overtreatment, thereby advancing precision medicine objectives.

Therefore, there is a critical need for effective recurrence prediction models. Existing histopathological scoring systems (e.g., PASS, GAPP, M-GAPP) exhibit suboptimal sensitivity and specificity (6, 7). Notably, the latest classification system groups pheochromocytomas (PHEOs) and sympathetic paragangliomas (PGLs) under a unified staging scheme. Recent analyses comparing historical data for PHEOs and PGLs (1, 2, 8–11) have revealed distinct recurrence risk profiles between these entities. This indicates that while prognostic factors for HNPGLs may overlap with those of systemic PGLs, they likely possess unique characteristics, underscoring the necessity for dedicated research to elucidate HNPGL-specific prognostic determinants.

TJPGLs is often carried out according to the Fisch or Glasscock–Jackson (12) classification system. To avoid the bias of Tympanic ball paragangliomas (TBPs) in Fisch A and B grades, all patients in this study were classified as glomus jugulare tumor in Fisch C and D grades, which are of glomus jugulare origin. This study analyzed clinical data from 318 patients, identifying independent predictors of recurrence and integrating them into a visual nomogram model. The model demonstrated robust predictive performance across training, validation, and test datasets. It provides clinicians with an intuitive and practical tool for individualized recurrence risk assessment. This facilitates the optimization of postoperative management strategies, including the identification of candidates for adjuvant RT and the design of tailored surveillance protocols, ultimately promoting precision medicine in the postoperative care of GJT patients.

## Materials and methods

This study retrospectively analyzed patients with GJT diagnosed in a tertiary medical centers between January 2003 and December 2022, confirmed by imaging and pathology. The inclusion criteria were: (1) Surgical treatment; (2) 2 years of follow-up data; (3) Complete records; (4) Glomus jugulare region; (5) Gross total resection (GTR: no visible tumor residue) or subtotal resection (STR: <5% residual tumor). The exclusion criteria were: (1) Preoperative radio/chemotherapy; (2) No surgery; (3) Incomplete data; (4) Partial resection; (5) Postoperative adjuvant therapy.

The 277 patients treated from January 2003 to December 2020 were randomly divided into a training set (70%, 194 patients) and a validation set (30%, 83 patients). The remaining 41 patients treated from January 2021 to December 2022 were assigned to the test set. All patients had a 2-year postoperative follow-up to monitor tumor recurrence. Ethical approval was granted by the Ethical Committee of a tertiary referral center.

For each patient, the following data were extracted from the database: (1) Patient characteristics, namely sex, age, clinical symptoms, disease duration, perioperative patient status, and intraoperative data. (2) Radiological data: computed tomography (CT) scans, magnetic resonance imaging (MRI) and digital subtraction angiography (DSA). (3) Intraoperative data: specifically operative time, blood loss, tumor growth patterns, and relationships with surrounding bone and nerves were assessed using imaging, surgical videos, and operative records. (4) Postoperative data: length of hospitalization, and DFS at ≥2 years from the date of surgery, were collected. All surgeries were performed by a single surgical team.

Categorical variables are expressed as frequencies and percentages, and quantitative variables as means ± standard deviations. Associations between variables and recurrence were analyzed using Chi-square or Mann-Whitney U tests (significance threshold: p ≤ 0.05) according to the data type. The identified factors were incorporated into a binary logistic regression model. Factors with a significance level less than 0.05 were included in the nomogram model construction. For variables that were not significant (i.e., with a significance level greater than 0.05), stepwise regression (bidirectional) was used to select modeling variables. For the binary logistic regression, the p-values, odds ratios(OR), and 95% confidence intervals (CIs) of independent risk factors were provided. The model fit was assessed using the Hosmer-Lemeshow goodness-of-fit test, and the model fit situation was observed.

Statistical analyses were performed using SPSS 25.0 (IBM SPSS Statistics) and R software (version 4.4.2). The t test was used to compare continuous variables, while the χ^2^ test was used for categorical variables. Univariable and multivariable logistic regression analyses were conducted to identify variables potentially associated with postoperative recurrence. Binary logistic regression was used for all predictive modeling of recurrence outcomes. Statistical significance was defined as P < 0.05. The predictive performance across the training set, test set, and validation set was evaluated using the area under the receiver operating characteristic curve (AUC-ROC). The statistical significance threshold for this study was set at p ≤ 0.05 (two-tailed).

## Results

This study enrolled a total of 318 patients meeting the inclusion criteria. All patients underwent GTR or STR during surgery and were followed up for over 2 years postoperatively. The recurrence criteria for PGLs were further refined as follows: local recurrence (disease in non-chromaffin-derived tissues within the primary tumor resection area), distant metastasis (disease in non-chromaffin-derived tissues outside the primary tumor region, such as bone metastasis), or new primary/uncertain (disease in chromaffin-derived tissues outside the primary tumor area, such as CBT or VGPL). Local recurrence is defined as pathologically confirmed tumor regrowth at the original site during post-operative follow-up, meeting growth rate criteria (volume increase ≥20% or annual diameter increase ≥2-3 mm) (5, 13). Based on these criteria, 68 patients (21.4%) experienced postoperative recurrence, with 3 patients undergoing surgical treatment more than three times. Among the entire cohort, the mean follow-up period was 8.7 years with a range of 2 to 22 years.

**Figure 1:**
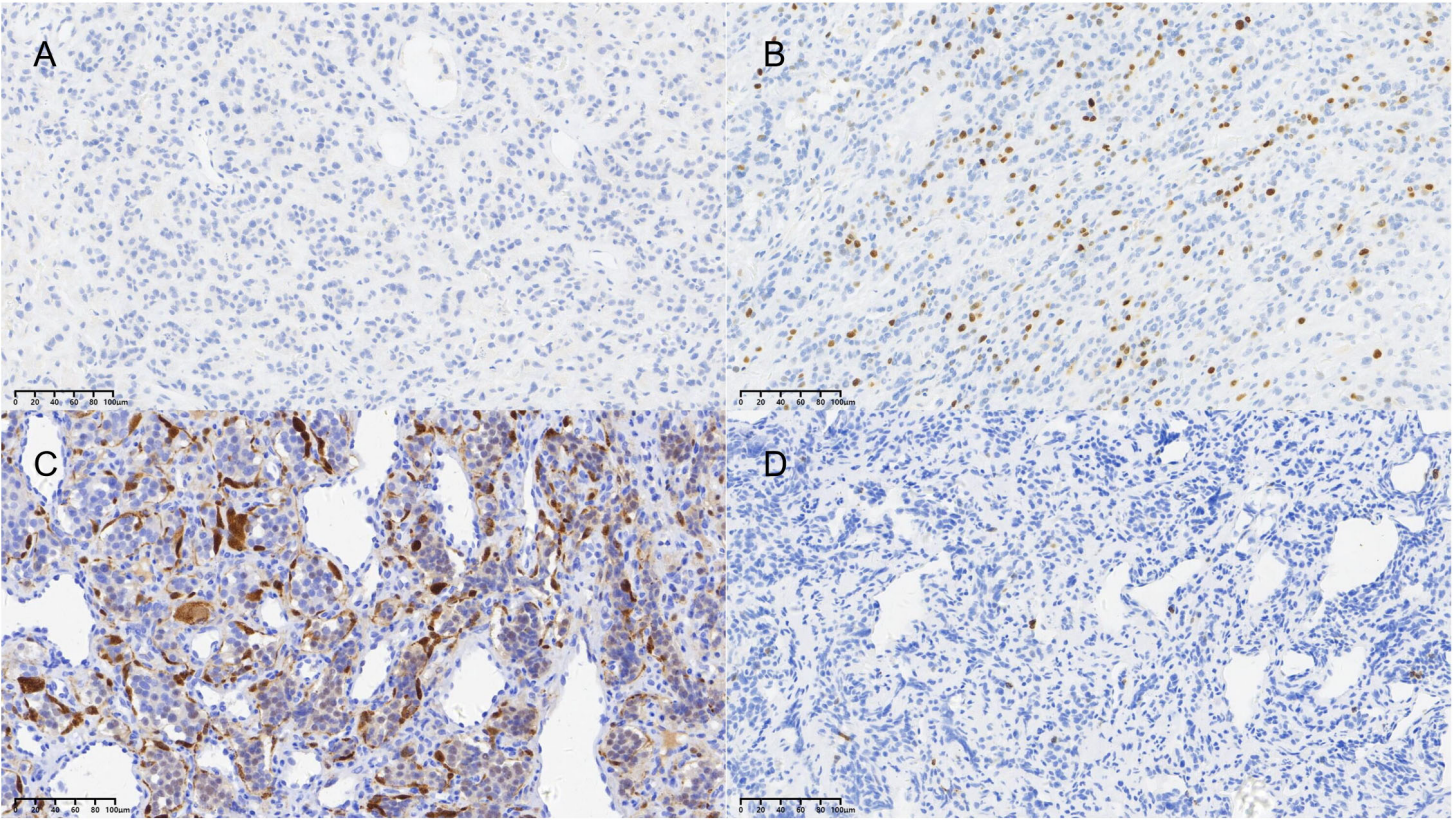
Representative immunohistochemical staining of key prognostic markers. **(A)** Positive nuclear staining for Ki-67 (brown chromogen, hematoxylin counterstain; original magnification ×20). **(B)** Positive staining for S-100 protein highlighting sustentacular cells (brown chromogen, hematoxylin counterstain; original magnification ×20). The graduated mark represents the actual magnification scale for the pathological tissue section.

Drawing on previous literature and considering the characteristics and anatomical specificity of GJT, potential factors influencing postoperative recurrence of systemic PGLs were screened and included in this study. Among the 194 patients in the training set, chi-square tests were performed for categorical variables and Mann-Whitney U tests for continuous variables. Variables with p ≤ 0.05 were seen as linked to recurrence. The clinical characteristics of patients are as follows. (**Table 1**) (**Figure 1**).

**Table 1:** Patients and tumor characteristics.

Characteristic		Training set	P
		n(%)	
Sex	Male	82(42.3)	0.109
	Female	112(57.7)	
Age		42.0±13.1 years	0.000
Immunohistochemistry			
	Syn	191(98.4)	1.000
	S-100	173(89.4)	0.000
	CgA	163(71.1)	0.470
	CD31	107(55.2)	0.688
	Ki-67	5.3±5.4	0.000
	CD34	103(53.1)	1.000
	CK	8(4.1)	0.655
	MelanA	48(24.7)	1.000
	Vimentin	165(85.1)	0.173
Symptoms			
	Hearing loss	131(67.5)	1.000
	Facial paralysis	54(27.8)	0.004
	Tinnitus	68(35.1)	0.184
	Ear fullness	19(9.8)	0.286
	Ear bleeding	30(15.5)	1.000
	Vertigo	14(7.2)	0.121
	Pulsatile tinnitus	104(53.6)	0.530
	Tongue deviation	12(6.2)	1.000
	Mass	65(33.5)	1.000
	Pain	39(20.1)	1.000
	Purulent discharge	34(17.5)	0.406
	Hoarseness	31(16.0)	0.050
	Dizziness	23(11.9)	0.812
	Choking cough	27(13.9)	0.023
	Duration of onset	48.9±63.2 months	0.456
Tumor Invasion Sites			
	Facial nerve	61(31.4)	0.000
	Internal carotid artery	90(46.6)	0.000
	Jugular foramen destruction	118(60.8)	0.014
	Mastoid	113(58.2)	0.053
	Tympanic cavity	129(66.5)	0.132
	Intracranial metastasis	37(19.2)	0.000
	Parotid gland	1(0.5)	1.000
	Lower cranial nerves	45(23.2)	0.851
	Infratemporal fossa	7(3.6)	0.108
	External auditory canal	27(13.9)	0.067
	Internal auditory canal	9(4.6)	0.284
Intraoperative Parameters			
	Intraoperative blood loss	1527.9±1974.9 ml	0.098
	Operative time	8.9±2.3 hours	0.332

Categorical variables were compared using Chi-square test; continuous variables were compared using Mann-Whitney U test;Refer to the comparative figure with **Figure 1** for cut-off values.

Univariate and binary logistic regression analyses were performed. Univariate analysis of 194 patients identified variables with P < 0.05, which were subsequently included in the logistic regression analysis. The results demonstrated that immunohistochemistry findings, clinical symptoms, and tumor invasion patterns were significantly associated with postoperative recurrence. (**Table 2**).

**Table 2:** Multivariate Analysis of Factors Associated with Postoperative Recurrence.

Variables	P	OR	95.0% CI for Exp (B)	
			Upper limit	Lower limit
Age	0.002	0.945	0.980	0.910
S-100	0.001	0.132	0.431	0.040
Ki-67	0.007	1.110	1.197	1.030
Facial paralysis	0.911	0.949	2.383	0.378
Choking cough	0.557	1.487	5.587	0.396
Hoarseness	0.097	2.805	9.947	0.829
Facial nerve	0.000	6.879	19.515	2.425
Internal carotid artery	0.030	2.756	6.887	1.103
Jugular foramen destruction	0.387	0.635	1.774	0.228
Intracranial metastasis	0.005	4.138	11.202	1.529

Multivariate logistic regression analysis was performed to identify independent predictors.

Stepwise regression (forward and backward) further optimized variable selection for modeling, identifying the final independent variables included in the model. (**Table 3**).

**Table 3:** Final Variables Incorporated into the Nomogram Model.

Variables	P	OR	95.0% CI for Exp (B)	
			Upper limit	Lower limit
Age	0.004	0.949	0.983	0.915
S-100	0.001	0.139	0.438	0.044
Ki-67	0.005	1.105	1.185	1.031
Facial nerve	0.000	4.528	10.276	1.995
Internal carotid artery	0.013	2.951	6.948	1.253
Intracranial metastasis	0.002	4.319	10.920	1.708

**Figure 2:**
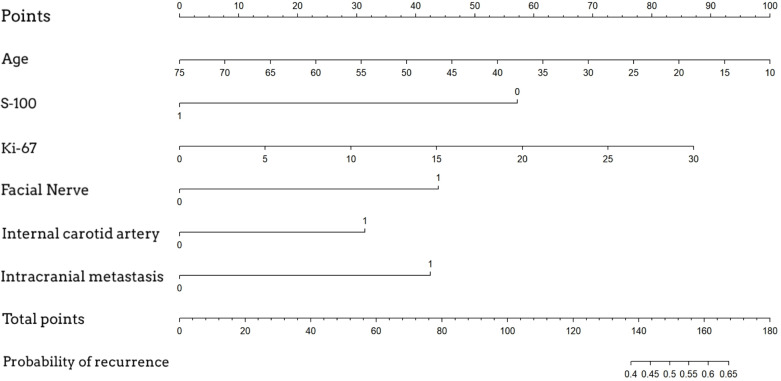
Nomogram for predicting postoperative recurrence of glomus jugulare tumors. To use the nomogram, locate the patient's value for each variable, draw a line upward to the 'Points' axis to determine the score for each variable, sum all the points, and then locate the total points on the 'Total Points' axis. A line drawn downward to the 'Risk of Recurrence' axis will indicate the individual's estimated probability of recurrence.

**Figure 3:**
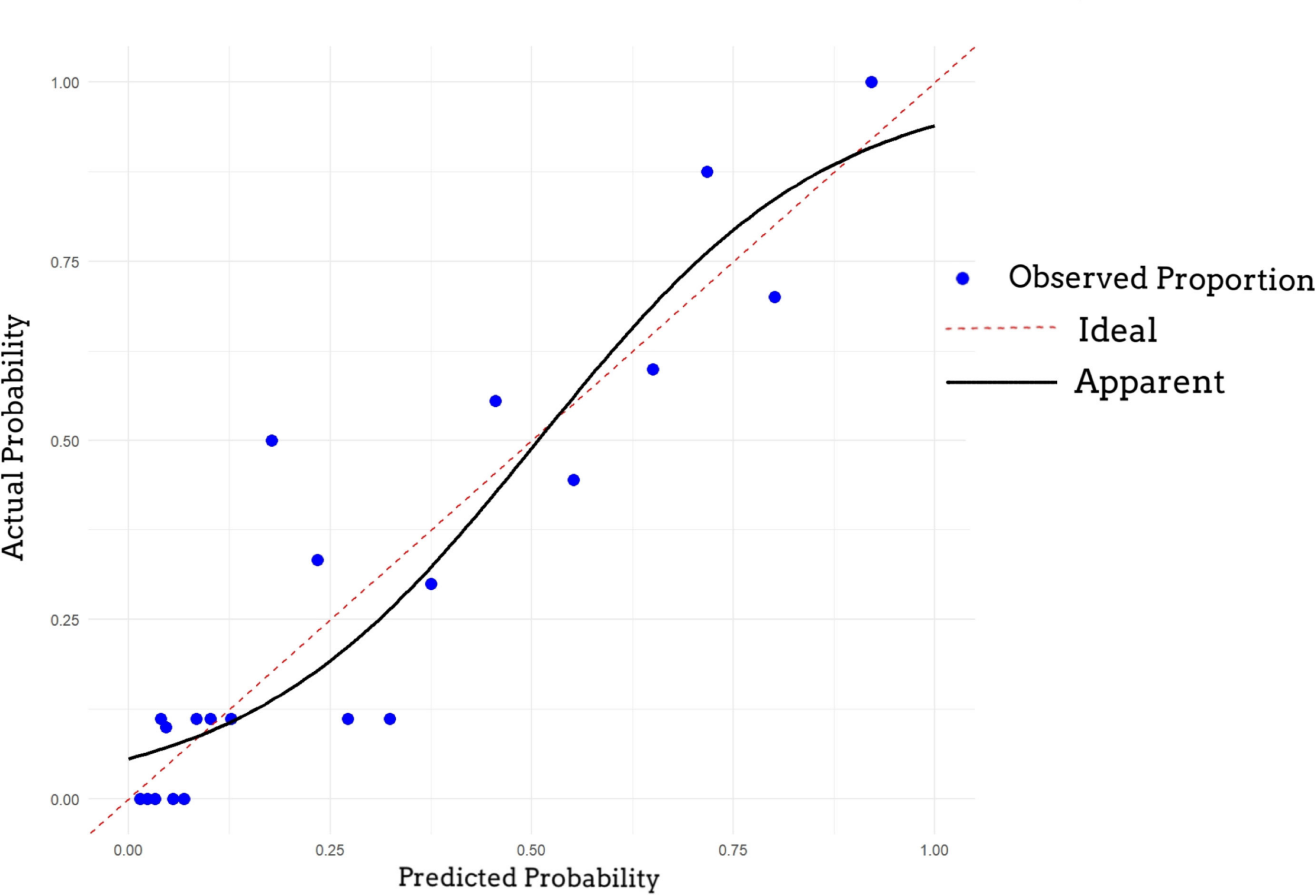
Calibration curve of the nomogram. The 45-degree dotted line represents the ideal prediction. The solid line represents the performance of the nomogram, where a closer fit to the diagonal indicates better calibration between predicted probability and observed outcome.

A nomogram was established based on logistic regression analysis, showing the weight of each included variable as a prognostic factor for recurrence in GJT patients after surgery. (**Figure 2**) In the nomogram, age and Ki-67 are included as continuous variables, while tumor invasion sites are incorporated as categorical variables. Patients with intracranial metastasis and negative S-100 expression had significantly higher odds of recurrence. The calibration plots demonstrated satisfactory consistency between the nomogram-predicted and actual observed values (**Figure 3**). The model showed good performance in both low-probability and high-probability predictions. Moderate deviations between observed and predicted probabilities were noted in the intermediate probability range (approximately 0.3-0.7).

The data of various influencing factors from the validation and test sets (**Table 4**) are incorporated into the model for validation.

**Table 4:** Number of Positive Cases or Mean Values of Predictive Factors in the Validation and Test Sets.

Variables	Validation set	Test set
Age	42.1±14.6 years	44.3±11.9 years
S-100	73(88.0)	38(92.7)
Ki-67	5.0±4.3	6.5±6.4
Facial nerve	37(44.6)	23(56.1)
Internal carotid artery	29(34.9)	18(43.9)
Intracranial metastasis	16(20.3)	10(24.4)

**Figure 4:**
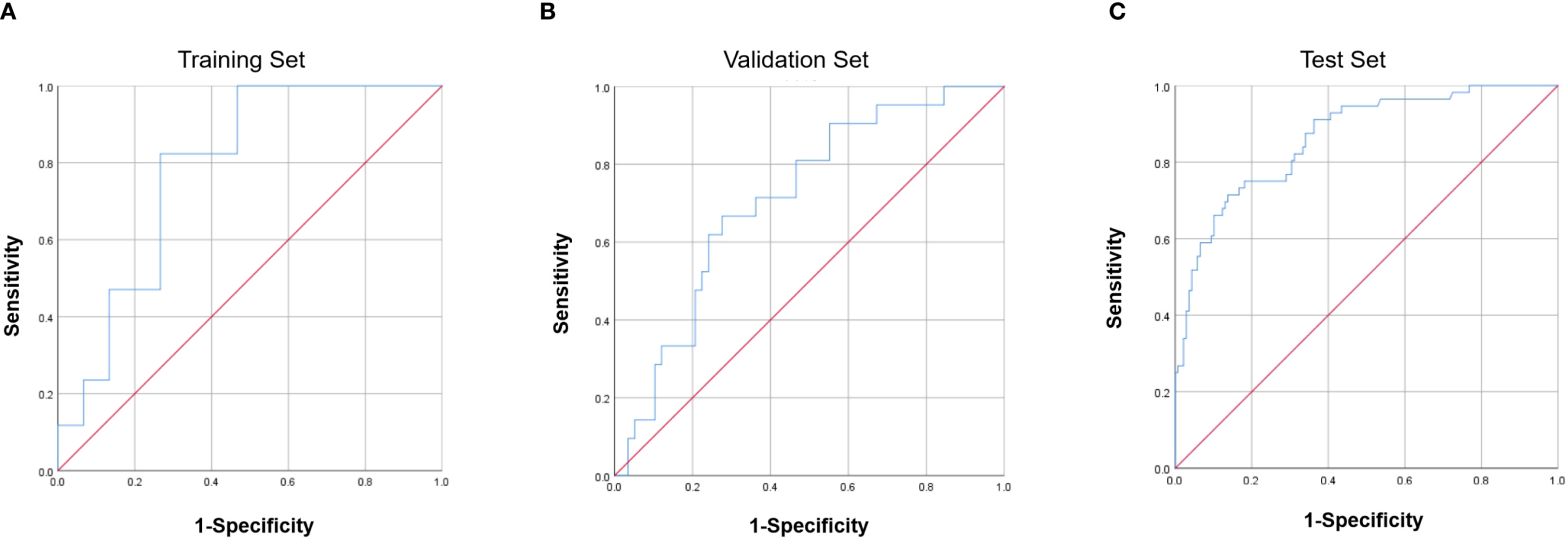
Receiver operating characteristic (ROC) curves of the nomogram. **(A)** Training set (AUC = 0.863). **(B)** Validation set (AUC = 0.711). **(C)** Test set (AUC = 0.784). AUC, area under the curve.

The ROC curves demonstrated acceptable predictive accuracy for postoperative recurrence in the training set (AUC: 0.863; **Figure 4A**), validation set (AUC: 0.711; **Figure 4B**), and test set (AUC: 0.784; **Figure 4C**).

## Discussion

In 2017, the World Health Organization (WHO) renamed the “malignant” nomenclature as “metastatic”, and the terms “benign” and “malignant” were abandoned. The most recent 2022 WHO classification suggests treating all tumors as malignant lesions with variable metastatic potential (14), highlighting their aggressiveness and high risk of recurrence.

This study is the first to conduct large-scale data collection and systematic analysis of patients treated with gross total resection or near-total resection alone. We comprehensively summarized disease-related therapeutic factors, developed a recurrence-predicting nomogram model based on prognostic predictors, and validated its stability and accuracy using an independent validation set and an test dataset. These findings provide actionable insights to guide clinical decision-making in the management of GJT.

With a mean follow-up of 8.7 years post-surgery, this study observed a recurrence rate of 21.4% demonstrated the highest recurrence risk among HNPGL, aligning with established findings. Among recurrent cases, 3 patients underwent surgery alone more than three times, with 4 developing local or distant metastases detected during subsequent treatments.

Our analysis identified age, immunohistochemical markers (S-100 and Ki-67), tumor invasion of the facial nerve or internal carotid artery, and intracranial metastasis as independent predictors of postoperative recurrence. Notably, the tumor invasion site showed significant associations in both univariate and multivariate analyses and contributed high weight in the nomogram model.

Studies on systemic PGLs (15) and GJT have shown a significant correlation between younger age and increased risks of both local recurrence and distant metastasis. In the study by Richter et al. (16) age was dichotomized at 42 years as a predictive factor, revealing elevated recurrence probabilities in patients younger than 42, though youth itself was not an independent predictor of recurrence. Consistent with these findings, our study confirms that younger patients are more susceptible to postoperative recurrence and incorporates age as a continuous variable in the nomogram.

Previous studies predominantly incorporated tumor volume as a predictor of recurrence. However, the complexity of anatomical locations and inherent inaccuracies in manual measurements may confound research outcomes and model development. To address these limitations, our study focused on surgically relevant factors by integrating tumor invasion sites into the predictive framework, thereby minimizing measurement bias and establishing a more direct correlation with intraoperative decision-making. The nomogram revealed that patients with intracranial invasion, carotid artery adhesion, or facial nerve invasion remained at high recurrence risk even after near-total resection. Facial nerve involvement emerged as secondary predictors. We hypothesize that preoperative irreversible neural deficits (House-Brackmann grade VI facial paralysis) may prompt surgeons to resect or reconstruct severely adherent nerves, whereas preserved preoperative function (House-Brackmann grade II-III facial weakness) often leads to nerve preservation despite intraoperative adhesions. This conservative approach risks residual superficial tumor tissue (“near-total resection”), potentially elevating recurrence likelihood.

Carotid artery adhesion is one of the factors affecting recurrence and is directly related to intraoperative bleeding. If not properly managed, it may lead to severe intracranial ischemic lesions in patients with poor contralateral blood supply compensation after surgery, resulting in serious consequences such as hemiplegia. Therefore, it is essential for patients to undergo preoperative angiography. According to the study by Andrea Bacciu et al (17), preoperative endovascular intervention, in selected cases, facilitates gross total tumor removal and significantly reduces the risk of intraoperative ICA injury. For these highly vascularized lesions, preoperative carotid artery stenting or permanent preoperative balloon occlusion is a key step (18). However, massive bleeding from the carotid artery and its branches during tumor resection remains a key factor in severe surgical complications and mortality.

In this study, Ki-67 emerged as a significant predictor of recurrence in GJT. Existing research on PGLs presents conflicting perspectives. For instance, Thompson et al. (6) argued that the Ki-67 index is not an isolated or independent predictive factor but interacts with clinical and histopathological features to collectively influence patient prognosisIn contrast, Yunying Cui et al. identified Ki-67 ≥3% as an independent predictor of pheochromocytoma and paraganglioma (PPGL) recurrence,Yiming Ding (19), in a smaller cohort, reported that Ki-67 exhibited excessive variability and lacked prognostic significance. In our study, among 52 patients with Ki-67 >10%, 22 experienced postoperative recurrence, and Ki-67 was significantly associated with recurrence risk (P = 0.008). These findings align with the widely adopted Pheochromocytoma of the Adrenal Gland Scaled Score (PASS) system—originally developed for systemic PGLs—which posits that Ki-67 reflects tumor proliferative activity and aggressiveness. Elevated Ki-67 indices may indicate heightened cellular proliferation, thereby increasing risks of recurrence or disease progression.

The S-100 protein family plays critical roles in cellular processes such as proliferation, apoptosis, differentiation, calcium ion homeostasis, and inflammation, and is implicated in various cancers (20). Deficiency of S-100 proteins can lead to tumor recurrence through multiple mechanisms, including upregulation of the fibroblast growth factor (FGF) pathway in tumor cells (21), alterations in cellular morphology and mechanical properties to enhance invasiveness, suppression of p53 function to promote proliferation and survival, and modulation of calcium ion balance, which may disrupt physiological functions such as cell adhesion, migration, and proliferation (22). Previous studies emphasize (23), that sustentacular cells in PGLs typically express S-100 and constitute a structural component of these tumors. Notably, as tumor malignancy increases, S-100 protein expression in sustentacular cells often becomes negative, indicating enhanced invasive potential. Researchers such as Charlie (24), Ling-Ling Wang (25), Caipu (23) have incorporated S-100 expression into scoring systems for evaluating PGLs recurrence risk. The findings of this study align with prior research, suggesting that the mechanism by which S-100 loss drives tumor recurrence in GJT may parallel that observed in PGLs at other anatomical sites. However, further experimental validation is required to confirm these molecular pathways.

Based on the analysis of the aforementioned predictive factors, the independent variables were incorporated into a nomogram model to develop a clinically applicable predictive model, and its performance was rigorously evaluated. As shown in **Figure 3**, the model demonstrated good fit with clinical data from the past 18 years and exhibited reasonable accuracy. However, the predictive accuracy for the recent 2-year testing dataset was slightly lower compared to the validation set, which may be attributed to the relatively short postoperative follow-up period. Specifically, the 41 patients in the testing set were newly diagnosed cases with a postoperative follow-up duration limited to 2 years. According to previous studies (26), the median time to postoperative recurrence is typically over 10 years, suggesting that the shorter follow-up period in this cohort may have influenced the model’s performance evaluation.

Compared to patients who received postoperative radiotherapy, who exhibit recurrence rates ranging from 0% to 18% (27–29), those treated with surgery alone show higher recurrence rates in previous literature (30). A systematic review encompassing >2,740 patients (10) reported 89.1% efficacy with stereotactic radiotherapy. Therefore, it is recommended that patients identified by this model as having a recurrence risk undergo adjuvant radiotherapy to reduce postoperative recurrence. Additionally, the necessity of postoperative surveillance should be emphasized for high-risk patients, who must be informed that recurrence risks may persist for over 10 years. While some studies suggest (31) that frequent short-term follow-up has limited utility in detecting GJT recurrence, we recommend that high-risk populations adhere to the National Comprehensive Cancer Network (NCCN) and Endocrine Society guidelines: follow-up every 3–6 months for the first 2 years postoperatively, followed by annual evaluations to monitor for recurrence.

### Limitations

Our study has several limitations that should be acknowledged. First, as a retrospective single-center study, the data were derived from historical medical records and examinations, which are subject to recall bias and data incompleteness. Second, the follow-up duration for the testing cohort was significantly shorter than that of other comparative groups, necessitating further validation through multicenter prospective studies with extended follow-up periods. Finally, due to limited documentation in the collected patient records, our study could not incorporate genetic factors such as Succinate Dehydrogenase Complex Iron Sulfur Subunit B(SDHB) gene mutations into the model. Prior research highlights the critical role of SDHB mutations in evaluating local recurrence and distant metastasis (29, 32), and most systemic paraganglioma risk assessment models include SDHB status as a key factor in their scoring systems (33). In our dataset, only a small subset of patients underwent genetic testing, precluding multivariate analysis of SDHB mutations. As a result, this variable was excluded from the nomogram construction. It should be noted that integrating SDHB gene status as a predictor would likely alter the risk contributions of the other factors within the model.

## Conclusion

Our study identified Ki-67 expression, S-100 protein status, tumor location, and age as significant predictors of postoperative recurrence in GJT patients. We developed and validated a nomogram model to predict recurrence risk in GJT patients, demonstrating its clinical utility. For patients classified as high-risk by the model, adjuvant radiotherapy is recommended to reduce recurrence, and the necessity of rigorous postoperative surveillance should be emphasized. This model provides a practical tool to guide individualized clinical management and improve outcomes in GJT patients.

The COVID-19 pandemic may have impacted follow-up compliance and timing for some patients, particularly those in the test set (2020–2022), potentially affecting recurrence detection.

The clinical presentation and management of GJT can exhibit geographical variations influenced by genetic predisposition and environmental factors. As highlighted in a recent review by Palade et al. (34), the incidence and predominant location of these tumors may differ across populations, which is a crucial consideration for the generalizability of prognostic models. Our study, derived from a single tertiary center’s experience, identifies a set of robust predictors. However, their relative weight and the model’s overall performance should be validated in international, multi-ethnic cohorts to ensure broad clinical applicability.

## Data Availability

The original contributions presented in the study are included in the article/supplementary material. Further inquiries can be directed to the corresponding author.

## Glossary

AUC: Area Under the Curve
CBT: Carotid Body Tumor
CI: Confidence Interval
CgA: Chromogranin A
CK: Cytokeratin
CT: Computed Tomography
DFS: Disease-Free Survival
DSA: Digital Subtraction Angiography
GAPP: Grading of Adrenal Pheochromocytoma and Paraganglioma
GJT: Glomus Jugulare Tumor
GTR: Gross Total Resection
HNPGL: Head and Neck Paraganglioma
ICA: Internal Carotid Artery
M-GAPP: Modified Grading of Adrenal Pheochromocytoma and Paraganglioma
MRI: Magnetic Resonance Imaging
NCCN: National Comprehensive Cancer Network
OR: Odds Ratio
PASS: Pheochromocytoma of the Adrenal Gland Scaled Score
PHEO: Pheochromocytoma
PGL: Paraganglioma
PPGL: Pheochromocytoma and Paraganglioma
ROC: Receiver Operating Characteristic
RT: Radiotherapy
SDHB: Succinate Dehydrogenase Complex Iron Sulfur Subunit B
STR: Subtotal Resorption
TBPs: Tympanic ball paragangliomas
TJPGL: Tympanojugular Paraganglioma
VGPL: Vagal Paraganglioma
WHO: World Health Organization
